# Study on the Novel High Manganese Austenitic Steel Welded Joints by Arc Welding for Cryogenic Applications of LNG Tanks

**DOI:** 10.3390/ma16062381

**Published:** 2023-03-16

**Authors:** Shuchang Zhang, Honghong Wang, Yangwen Wang, Liang Cao

**Affiliations:** 1 College of Science, Wuhan University of Science and Technology, Wuhan 430081, China; 2 The State Key Laboratory of Refractories and Metallurgy, Wuhan University of Science and Technology, Wuhan 430081, China; 3 Jiangsu Watts Energy & Engineering Co., Ltd., Nantong 226200, China

**Keywords:** high-Mn austenitic steel, welded joint, similar welding consumable, mechanical property

## Abstract

The novel high-Mn austenitic steel is becoming a promising steel for cryogenic applications of LNG tanks. The welded joints take a critical role in cryogenic service for storage tanks. In this work, we developed well-matched high-Mn welding consumables and prepared the welded joints by shielded metal arc welding (SMAW), submerged arc welding (SAW) and gas tungsten arc welding (GTAW). The detailed welding parameters were proposed first, then the welding quality, mechanical properties, and microstructure were investigated. The results show that good welding quality, excellent mechanical properties, and stable levels of mechanical properties were obtained for high-Mn steel welded joints using similar welding consumables, the solid core of electrodes, and solid welding wires. Notably, the lowest cryogenic absorbed energy was found at 5 mm away from the fusion line rather than at the fusion line. The hardness of the welded joints was detected to be less than 280 HV due to the whole austenitic microstructure.

## 1. Introduction

The growing global demand for liquefied natural gas (LNG) as an environmentally friendly energy source and the increasing quantity of construction and operation of LNG-fueled ships create a growing demand for cryogenic steels [1,2,3]. Owing to the excellent comprehensive properties at cryogenic temperature and room temperature, as well as the low cost [4,5,6], the high manganese austenitic cryogenic steel (high-Mn steel) has attracted wide attention recently [7,8]. A tremendous amount of existing research reported that high-Mn steel can meet the requirements of cryogenic service of cargo and fuel tanks of LNG carriers and LNG-fueled ships [9,10,11,12,13,14,15].

Welding is a key technology for high-Mn austenitic cryogenic steel to build the cargo and fuel tanks of LNG carriers and LNG-fueled ships [16]. The mechanical properties of welded joints (WJ) are a critical case that determines the integrity and safety of cargo and fuel tanks of LNG. According to the Interim Guidelines on the Application of High Manganese Austenitic Steel for Cryogenic Service [17] issued by the International Maritime Organization (IMO) in 2019 and the Application Guidelines on High Manganese Austenitic Cryogenic Steel [18] issued by China Classification Society (CCS) in 2021, the requirements for the mechanical properties of high-Mn steel WJ used in LNG cargo and fuel tanks is specified, as shown in Table 1. It provides a standard reference for cryogenic application of high-Mn steel. In recent years, some works on high-Mn steel welding have been carried out, including laser welding [19,20], electric beam welding [21], laser-MIG hybrid welding [22] and other methods. However, some conventional welding methods such as shielded metal arc welding (SMAW), submerged arc welding (SAW), and gas tungsten arc welding (GTAW) are still widely used in the fabrication of LNG cargo and fuel tanks in China.

With respect to SMAW of high-Mn steel, J.K. Ren [23] and X.Y. Fan [24] provided the mechanical properties of dissimilar welded joints using the Ni-based welding electrode, due to the lack of high-Mn steel welding electrodes, which have met the requirement of IMO. However, the high price of Ni-based welding consumables has also become a problem during the construction of LNG storage tanks. In addition, regarding the dissimilar welding, element (C, Mn and Ni) diffusion and segregation occurred in the fusion zone due to the composition difference between the base metal (BM) and the weld metal (WM). The C-Mn-Si segregation zone caused inhomogeneity of microstructure and hardening of austenite matrix, which affected the plastic deformation ability and finally reduced the cryogenic impact toughness of the heat-affected zone (HAZ).

For the SAW of high-Mn steel, X.Y. Fan [24] also investigated the SAW welded joint with Ni-based welding consumables. The tensile strength and elongation of dissimilar welded joints at room temperature was 713 MPa and 24%, respectively, while the impact absorbed energy of WM, fusion line (FL), and HAZ at −196 °C was only 9 J, 32 J, and 15 J, respectively. Compared with the dissimilar SMAW welded joint, the lower value of cryogenic impact toughness for SAW welded joints was presented in the HAZ. It was found that the cryogenic impact toughness of welded joint by dissimilar welding consumables was significantly worse. Gyubaek An et al. [25] used the undermatching SAW welding consumables to weld the high-Mn steel plate. The tensile strength of the welded joint at room temperature was 743 MPa and the tensile strength was 818 MPa at −165 °C. The results of tests indicated that the base metal and the welded joint satisfied the IGC/IGF code for each requirement. However, the specific information of the welding consumables was not reported.

For the GTAW of high-Mn steel, Kim et al. [26] fabricated a bell-shaped storage tank for a thermal fatigue test with GTAW and flux core arc welding (FCAW). Under the condition that the internal pressure was kept at ambient pressure, the thermal cycling experiment was conducted on the tank with a colling medium of liquid nitrogen. The result of radiographic tests showed that there were no defects after 50 cycles. However, the relevant mechanical properties of welded joint were not reported in this paper. Additionally, the flux-cored wire was strongly affected by the filling rate and the uniformity of powder distribution. It is quite possible that the WM formed an inhomogeneous composition, leading to unstable mechanical properties [27]. Therefore, it is important to develop the solid wires of welding consumables to match the high-Mn steel.

Even now, the work of high-Mn steel-welded joints by conventional arc welding methods using similar welding solid wire consumables, meeting conventional standards, and providing detail information on welding parameters and welding consumables has not been reported yet. In order to provide valuable data for the application of high manganese steel in LNG storage tanks, the SMAW with the solid electrode core, SAW with the solid welding wire, and GTAW with the solid welding wire were developed and conducted in this study. The mechanical properties were studied mainly on the welded joints using different welding methods and parameters.

## 2. Experimental Material and Procedure

### 2.1. Materials

The base materials are high-Mn steel plates in thicknesses of 10 mm and 30 mm. They were fabricated by hot-rolling at 1100–920 °C after homogenizing at 1200 °C for 200 min and cooling to room temperature at 10 °C/s. The chemical composition and the mechanical proprieties in the transverse direction of the base material are presented in Table 2 and Table 3, respectively. The microstructure of the base material is full austenite.

### 2.2. Welding Procedure

**Figure 1:**
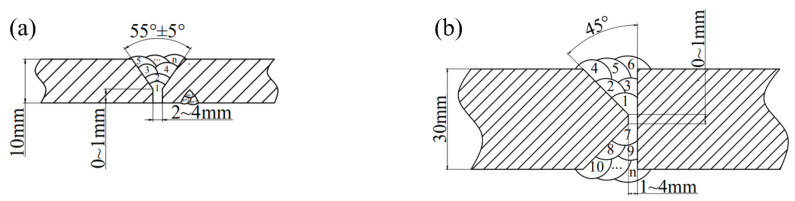
Size of V-type (**a**) and K-type (**b**) groove and weld bead distribution.

Ten plates 1000 mm × 150 mm × 10 mm and four plates 1000 mm × 150 mm × 30 mm were processed by plasma cutting. As shown in Figure 1, the V-type (thickness of 10 mm) and K-type (thickness of 30 mm) groove along the length direction were machined on the cleaned plates.

For SMAW, the solid core electrode with diameters of 3.2 mm and 2.5 mm, named as JMn25, were used for butt weld in the flat position (1G), vertical position (3G), and overhead position (4G). The SMAW samples were designated as SM-1, SM-3, and SM-4, respectively. For GTAW, the solid wire with a diameter of 2.5 mm, named TGMn25, was used with the shielded gas of argon with a purity of 99.99% for butt weld in the flat position. The GTAW sample was designated as GT-1. For SAW, the solid wire with a diameter of 3.2 mm, named MHMn25, was used for butt weld in the flat position. The SAW samples were designated as SA-1, SA-1-15, and SA-1-30, respectively.

**Figure 2:**
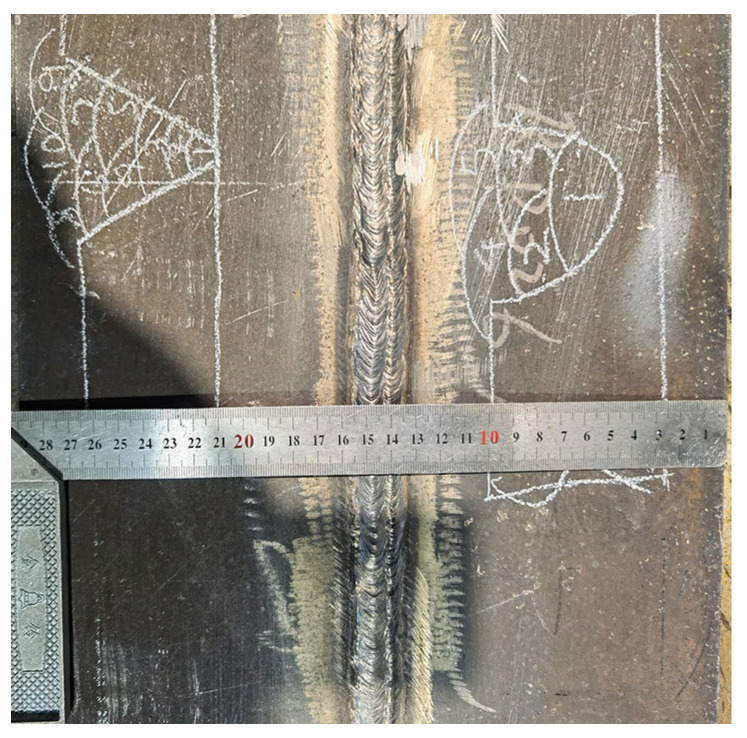
Appearance of the SM-1 test plate.

The detailed welding parameters are shown in Table 4. The inter-pass temperature of below 150 °C was performed during the welding process. The root pass was cleaned with carbon-arc gouging. The current and voltage of backing welding is slightly lower than that of filling and cover welding. Figure 1 diagrams the weld bead distribution. The multi-pass and multi-layer welded joints were prepared with the welding heat input of 11~32.5 kJ/cm. The SM-1 test plate is shown in Figure 2.

### 2.3. Experimental Procedure

#### 2.3.1. Chemical Composition Analysis of Weld Metal

**Figure 3:**
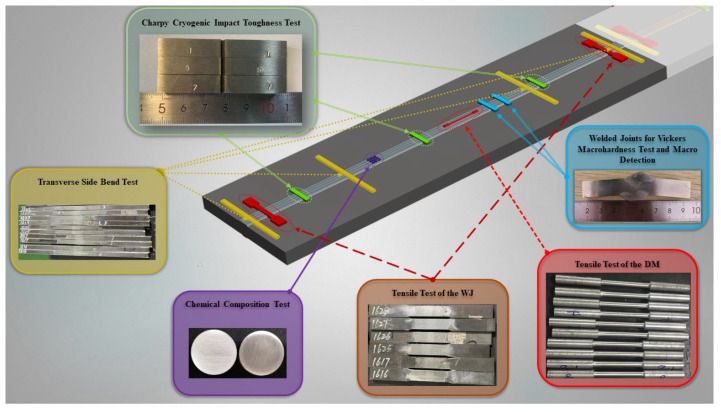
Schematic diagram of experimental sample (WJ: welded joint, DM: deposited metal).

As shown in Figure 3, the cylindrical samples with the size of Ф 5 mm × 2 mm and a weight of 4 g were obtained from the weld bead. An inductive coupled plasma emission spectrometer (ICP) with the model of Agilent ICP-OES 725 ES was used to detect the chemical composition of the weld metal.

#### 2.3.2. Nondestructive Test (NDT) of Welding Quality and Macro Inspection of the Welded Joint

According to the Application Guidelines on High Manganese Austenitic Cryogenic Steel by CCS, the NDT of welding quality is required. The surface penetration and X-ray flaw detection tests were conducted within 24 h of the completion of welding. According to ISO 17639:2003 (E), macro inspection was carried out on the welded joints to detect cracks, pores and other defects.

#### 2.3.3. Tensile Properties at Room Temperature

As shown in Figure 3, one rod-shaped tensile specimen of the weld metal and two plate-shaped tensile specimens of the welded joints were taken from each test plate. For the weld metal, the diameter of the parallel section for the rod-shaped tensile specimen was 8 mm and the tensile test was carried out on an MTS CMT5305 machine according to ISO 5178:2001 standard with a strain rate of 0.00125 s^−1^. For the welded joints, the width of the parallel section for plate-shaped tensile specimens was 25 mm, and the tensile test was carried out on an MTS SHT4106 machine according to ISO 4136:2001 standard with a strain rate of 0.00125 s^−1^.

#### 2.3.4. Charpy V-Notch Impact Test of the Welded Joint at −196 °C

**Figure 4:**
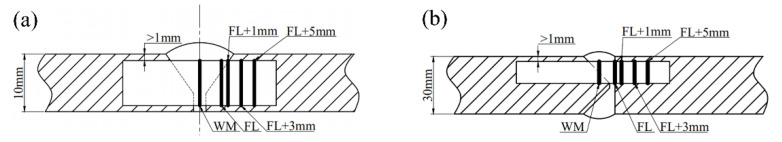
V-notch location of the sample: (**a**) thickness of 10 mm; (**b**) thickness of 30 mm.

Toughness is usually measured through the Charpy V-notch impact test in the experiment of the welding process. Three sizes of standard Charpy V-notch specimens (5 mm × 10 mm × 55 mm, 7.5 mm × 10 mm × 55 mm, and 10 mm × 10 mm × 55 mm) were prepared in this work depending on the thickness of test plates. Charpy V-notches were located at the center of the weld metal, FL, FL + 1 mm, FL + 3 mm, and FL + 5 mm, as shown in Figure 4. The cryogenic impact test of the welded joint at −196 °C was carried out on an MTS ZBX2302-EC pendulum impact testing machine according to ISO 9016:2001 standard.

#### 2.3.5. Side Bending Test of the Welded Joint at Room Temperature

Four side bending test samples with a thickness of 10 mm were cut from each welded joint. The side bending test was carried out on the automatic steel bar bending test machine with a power of 2.2 kW according to ISO 5173:2000. The samples were bent to 180° by a bending mold with a diameter of 40 mm.

#### 2.3.6. Vickers Hardness Test

**Figure 5:**
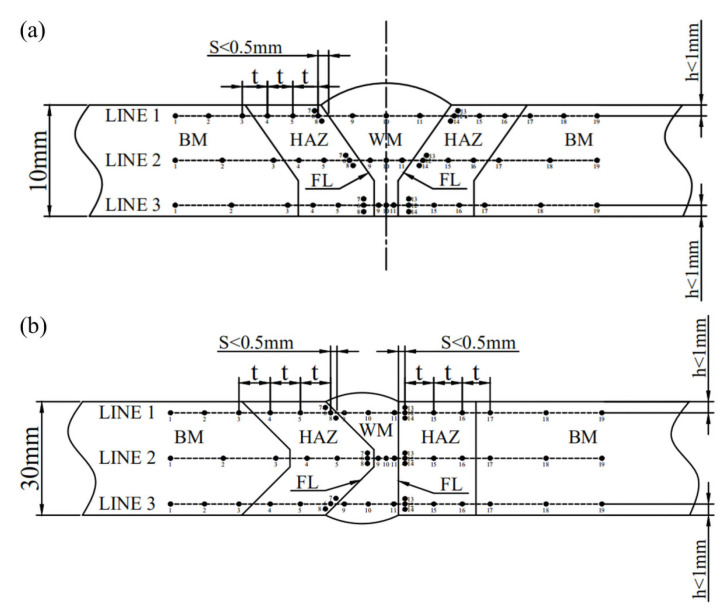
Point distribution of the hardness test of the welded joint: (**a**) thickness of 10 mm; (**b**) thickness of 30 mm.

The transverse sections of the welded joints were prepared and etched with 10% nitric acid alcohol for 15 s. The Vickers hardness was measured on the top, middle, and bottom, as shown in Figure 5. Innovatest Falcon 500 Vickers hardness tester (Maastricht, The Netherlands) was used with a load of 10 kg according to ISO 9015-1:2001 standard.

#### 2.3.7. Microstructure Observation

The samples for microstructure observation of SMAW, GTAW, and SAW welded joints were prepared. They were etched with 10% nitric acid alcohol for 25 s first, and then with saturated Na_2_S_2_O_3_ solution for 15 s. The Carl Zeiss AXIO optical microscope was used for observation microstructure.

## 3. Results

### 3.1. Chemical Composition of the Weld Metal

Table 5 shows the chemical composition of the investigated weld metals with different weld methods. The design criterion of the investigated high-Mn welding consumables is having a similar chemical composition with the high-Mn steel. Therefore, the main elements in Table 5, like C, Mn, Si and Cr, are similar to the base metal.

### 3.2. Examination of Welding Quality

**Figure 6:**
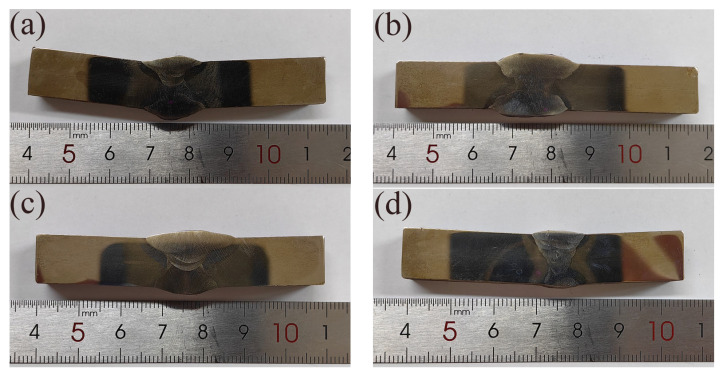
Macroscopic photographs of the welded joints: (**a**) SM-1; (**b**) SM-3; (**c**) SA-1; (**d**) GT-1.

**Figure 7:**
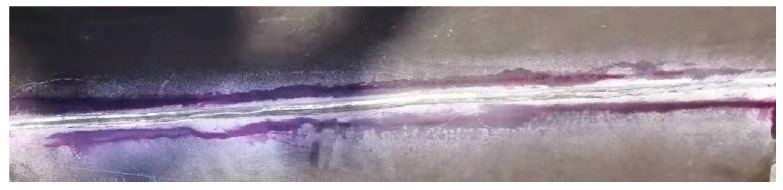
Surface penetration detection of the SM-1 welded joint.

The macroscopic photographs of four welded joints are shown in Figure 6. The surface penetration detection of SM-1 is shown in Figure 7. It was found that there were no defects by artificial visual inspection. The detection results for all the investigated weld joints are presented in Table 6. The macro appearance detection showed that the welding consumables were matched very well with the base metal. It is proposed that the high-quality welded joints can be obtained with similar consumables under the appropriate welding process and parameters.

### 3.3. Tensile Properties at Room Temperature

Table 7 presents the results of the tensile test of the weld metals and the welded joints. For SMAW weld metals, the yield strength was around 438~447 MPa and the tensile strength was around 717~760 MPa. The elongation decreased from 43.9% at 1G to 30.9% at 4G. It was known that the mechanical properties of 3G and 4G weld metal were slightly poor than the 1G weld metal due to the different weld pool shape and the different welding metallurgy [28]. It was the same for the welded joints, as shown in Table 7. The tensile strength of the 1G welded joints was 764 ± 9 MPa, which was better than that of 3G and 4G welded joints.

For GTAW sample, the yield strength of the weld metal was 469 MPa, the tensile strength of the weld metal was 758 MPa and the elongation was 50.3%. The tensile strength of the welded joints was 772 ± 8 MPa.

For SAW samples, the yield strength and tensile strength of the weld metals increased from 392 MPa to 549 MPa, and from 691 MPa to 882 MPa with the same welding heat input of 15 kJ/cm as the thickness increased from 10 mm to 30 mm. the elongation decreased from 51.5% to 44.5%. When the welding heat input increased to 30 kJ/cm at the plate with thickness of 30 mm, the yield strength of the weld metal increased to 565 MPa, the tensile strength decreased to 764 MPa, and the elongation also deceased to 33.0%, while the tensile strength of the welded joints remained around 754 ± 11 MPa.

### 3.4. Cryogenic Charpy Impact Toughness

It was found that large welding residual deformation occurred after welding of high-Mn steel. Therefore, the Charpy-V notch impact test specimens were machined in three sizes: 5 mm × 10 mm × 55 mm for SM-1 and SM-4; 7.5 mm × 10 mm × 55 mm for SM-3, GT-1, and SA-1; and 10 mm × 10 mm × 55 mm for SA-1-15 and SA-1-30.

**Figure 8:**
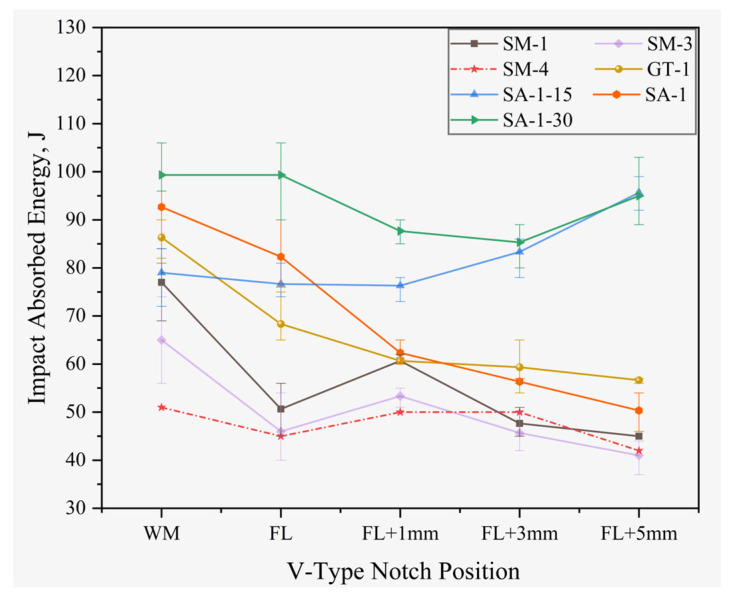
Cryogenic V-notch Charpy impact absorbed energy.

According to the Chinese Petroleum and Natural Gas Industry Standard SY/T6194-2003, the decreasing coefficient is 0.55 for the sample with a size of 5 mm × 10 mm × 55 mm, and is 0.8 for the sample with the size of 7.5 mm × 10 mm × 55 mm. The equivalent value and average value of absorbed energy at −196 °C are shown in Table 8 and Figure 8.

Table 8 shows that the impact absorbed energy at −196 °C for all investigated welded joints exceeded the standard value of 27 J. Notably, the absorbed energy of the weld metals exhibited a more stable value.

As for the welded joints at a 10 mm thickness, the highest average absorbed energy was detected at each weld metal center. They were 77 J, 65 J, 51 J, 86 J, and 93 J, respectively. The lowest absorbed energy was found at FL + 5 mm, as shown in Table 8. The average value was around 40~50 J.

For SMAW welded joints, the absorbed energy value at FL was lower than that at WM and FL + 1 mm, while the impact value continuously decreased from FL + 1 mm to FL + 5 mm. For GTAW and SAW welded joints, the impact value showed a continuous downward trend from WM to FL + 5 mm. In the 1G welding position, the cryogenic impact toughness of SAW welded joints was the best, followed by the GTAW welded joints. The poorest cryogenic impact toughness was seen in the SMAW welded joints. For the SMAW welding method, the cryogenic impact toughness of the 1G welding position was better than that of the 3G and 4G welding position, which was the same as the tensile results.

As for the welded joints for 30 mm thick plate, the highest average absorbed energy values of SA-1-30 sample were presented at WM and FL, at 99 J. The highest average absorbed energy value of SA-1-15 was presented at FL + 5 mm, at 96 J. With the increasing of heat input, from WM to FL + 3 mm, the cryogenic impact toughness of SAW welded joints was improved.

The mechanical properties are influenced by the welding parameters, such as groove angle, welding heat input, plate thickness and welding position. Although we got results with common welding parameters in this research, the strength-toughness mechanism was not extremely clear yet. Therefore, based on this work, a large number of studies are also needed to explore the weldability of high-Mn steel.

### 3.5. Transverse Side Bend Test of the Welded Joints

All welded joint specimens were taken from each welding plate for transverse side bend test at room temperature. As shown in Table 9, there was no opening defect in the investigated welded joints. This result showed good cracking resistance and good physical metallurgy deposition of the weld metal and the base metal using similar welding consumables.

### 3.6. Vickers Macrohardness Distribution

**Figure 9:**
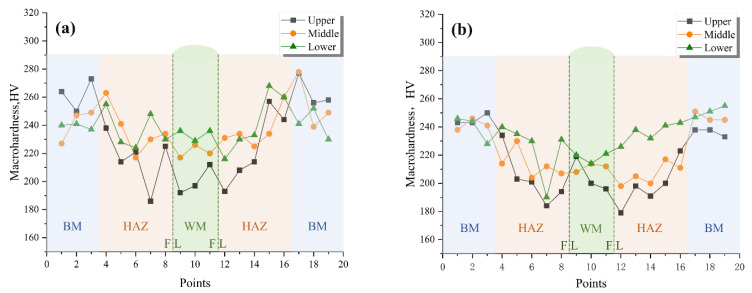

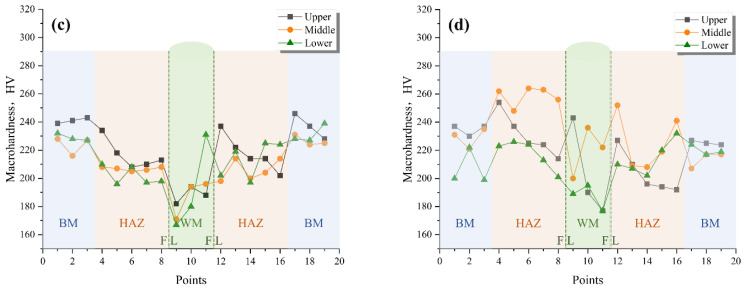
Vickers hardness of the welded joint: (**a**) SM-1; (**b**) SM-3; (**c**) SA-1; (**d**) GT-1.3.7. Microstructure.

The results of the Vickers macrohardness test showed that the welded joints had a Vickers hardness less than 280 HV. As shown in Figure 9, for SM-1, SM-3, GT-1, and SA-1, the highest hardness value was around 230–270 HV at the base metal. The lowest hardness value was around 240 HV at the weld metal. The hardness value of the HAZ was detected between the weld metal and the base metal.

In general, the hardness at the upper line was lower than that of middle and bottom line because the middle and bottom line were subjected to the more welding thermal cycles than that of the upper line. For the HAZ, some sharp drops of hardness were found. However, the locations were different in SMAW, GTAW and SAW samples. It was supposed to be influenced by the difference in welding heat input. More experimentation is needed to explore it further.

**Figure 10:**
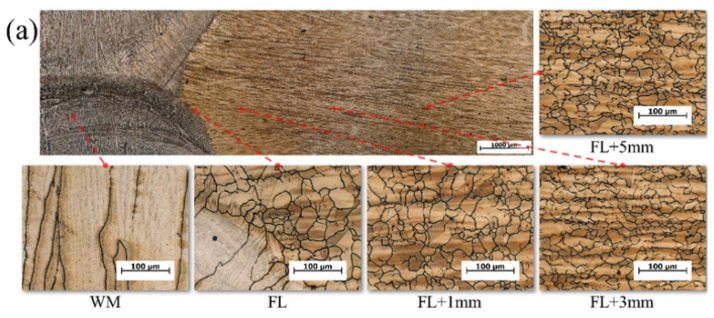

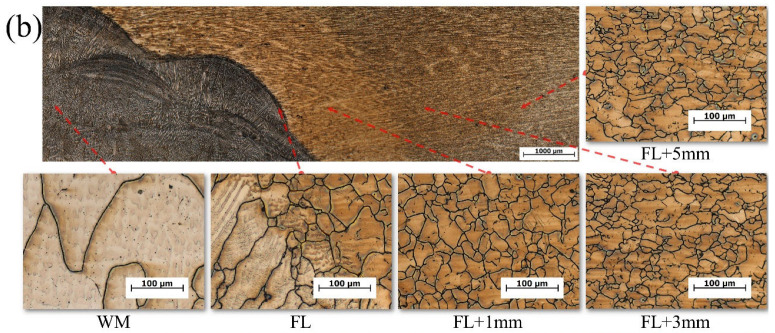

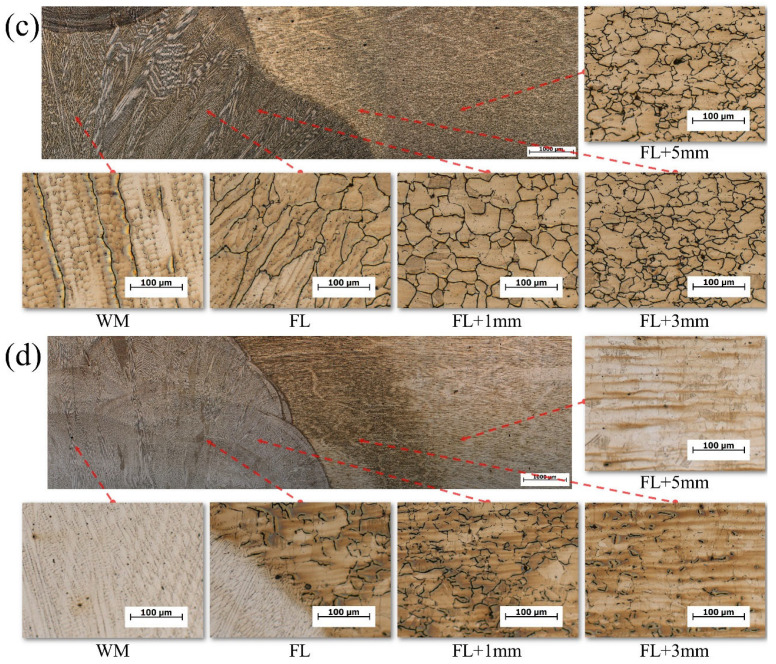
Microstructure of the welded joints: (**a**) SM-1; (**b**) SM-3; (**c**) SA-1; (**d**) GT-1.

Figure 10 shows the optical microstructure of the SMAW, GTAW, and SAW welded joints. It was found that the microstructure of all welded joints was composed of austenite. What was different was the morphology of the austenite. The base metal represented the equiaxed grain, due to the hot-rolling process. In the HAZ, some grains refined during recrystallization by welding thermal cycles and some grains coarsened by the peak temperature of 1320~1350 °C. The morphology of the HAZ was also equiaxed grain. The weld metal solidified based on the molten coarse grain and grew up in the columnar crystalline to the weld metal center. Due to the full austenitic, a hardness below 280 HV is reasonable.

The base metal had the hot-rolled strips in Figure 10. It was found that the enriched zone with Mn, C, and other elements, and the depleted zone were at regular intervals located in the hot-rolled strips. It was supposed to influence the weld metal solidification by the segregation of Mn, C and other elements during solidification. The microstructure of the weld metal was columnar crystalline which was extended from the fusion line to the weld metal center. The investigation of weld pool solidification and characterization of the weld metal of high-Mn steel will be submitted in another paper.

## 4. Discussion

### 4.1. The Excellent Mechanical Properties of Conventional Arc Welded Joints Obtained by Suitable Welding Parameters

The mechanical properties of the welded joints were excellent, Table 10 showed the mechanical properties of the welded joint and the weld metal. Compared with the value required in the specification, the higher strength, higher elongation, and higher cryogenic toughness are obtained in the present work, as shown in Table 10. All the welded joints reached a tensile strength exceeding 700 MPa. The hardness of all welded joints also met the requirements of less than 350 HV. The suitable welding parameters play an important role in the experimental process, especially the heat input. The heat input of 13~30 kJ/cm is recommended in practice.

For the plate with a thickness of 10 mm, the GTAW welded joint with the heat input of 13 ± 2 kJ/cm in the flat position had the best comprehensive mechanical properties. That was due to the shielded gas of argon, stabilizing the arc during the welding process, and refining the weld metal. For the plate with the thickness of 30 mm, the mechanical properties of SAW welded joints by the heat input of 15 ± 2 kJ/cm in the flat position was better than that of 30 ± 2.5 kJ/cm.

### 4.2. The Superior Cryogenic Toughness of HAZ by the Similar Welding Consumables

The impact toughness results of the welded joints (in Table 7) showed that the whole welded joints exhibited the superior cryogenic impact toughness. Specifically, the fusion line was not the poorest zone, which was quite different from the ferritic steels like low-alloy high-strength steels [29]. It was because that the weld metals were designed to have the similar chemical composition (in Table 5) with the base metal (in Table 2), which allowed the weld pool to solidify with the same phase of austenite and the weld metal not to undergo any phase transformations during continuous cooling [30]. Although the grain coarsened at FL, the coarse grain was fortunately not detrimental to cryogenic toughness for high-Mn steel [31].

On the other hand, the similar welding consumables for the high-Mn steel in this research prevented some bad issues from dissimilar welding. It had been reported that welding high-Mn steel using nickel-based weld consumables (matched 9Ni steel) worsened cryogenic impact toughness at the coarse grain heat-affected zone (CGHAZ) [23]. This was confirmed to attribute mainly to the difference of chemical composition between the weld metal and the base metal, which led to the twinning difficulty and the lower plastic deformation capacity [24]. Meanwhile, the hardened austenite developed a high defect density, hindered the dislocation migration, and finally worsened the cryogenic impact toughness in CGHAZ [23].

In addition, due to the different physical properties, such as the thermal conductivity and the thermal expansion coefficient of dissimilar metals, the weaker fatigue resistance of the welded joint was expected to occur commonly. This evidence should be paid more attention in the cryogenic service. The similar coefficient of thermal expansion may reduce the residual stress of welding during the solidification of the molten pool and improve the fatigue resistance of the welded joint.

### 4.3. The Strengthen of High-Mn Austenitic Weld Metal by Alloy Design

The weld metal was detected to have a lower strength than the base metal in this study because the base metal can strengthen via the rolling process and heat treatment. However, the weld metal only encounters solidification directly from liquid and the re-heating through the welding thermal cycle. In order to achieve high strength of high-Mn weld metal, the composition design of the weld metal is an effective way. It was well-known that the stacking fault energy (SFE) was the main factor in controlling deformation mechanisms of high-Mn steels, and it is mainly controlled by composition and temperature. In this work, the SFE was designed to be within the range of 20~40 mJ/m^2^ [9,32] to ensure the predominant TWIP effect. According to the Hall-Petch effect, the formation of mechanical twins evolves the creation of new crystal orientation and reduces the effective mean free path of dislocation [33]. The flow stress can be increased by the Hall-Petch effect, which can suppress the necking during the tensile deformation. In addition, it restrains crack propagation effectively during impact tests. Therefore, the strength, ductility, and toughness of high-Mn weld metal can be improved by the TWIP effect at the same time [9]. According to our previous research, the SFE of the investigated high-Mn steel decreased from 24.1 mJ/m^2^ at 20 °C to 17.1 mJ/m^2^ at −196 °C [4].

It had been reported that the increased Ni content by 1 at. % resulted in the increase of SFE by 10 mJ/m^2^, while the increased Cr and Si content by 1 at. % resulted in the decrease of SFE by 1.7 mJ/m^2^ and 4 mJ/m^2^, respectively [34]. Thus, adding an amount of Ni and reducing the content of Cr and Si to the weld metal was designed to improve SFE and to enhance TWIP effect.

Calculating the stacking fault energy of designed weld metal is in the proposed following formula [4]:

(1) $${\mathrm{SFE}=2\rho \Delta G}^{\gamma \to \varepsilon }{+2\sigma }^{\gamma /\varepsilon }$$

Here, ${\Delta G}^{\gamma \to \varepsilon }$ represents the calculated Gibbs energy change related to chemical composition and temperature, ρ represents the Molar surface density along {111} plane, and $\sigma^{\gamma /\varepsilon }$ is the interface energy between γ and ε phases. Some research [35,36] showed the interface energy of TWIP steel was normally 10 mJ/m^2^. To determine the value of ρ and ${\Delta G}^{\gamma \to \varepsilon }$ the following Formulas (2) and (3) are used:

(2) $$\rho =\frac{4}{\sqrt{3}}\frac{1}{a^{2}N}$$

(3) $${\Delta G}^{\gamma \to \varepsilon }=\sum x_{i}{\Delta G}_{i}{}^{\gamma \to \varepsilon }+\sum x_{\mathrm{Fe}}x_{j}\Omega_{\mathrm{Fej}}^{\gamma \to \varepsilon }$$

Here, $x_{i}$ indicates the Mole friction of all the elements in steel, ${\Delta G}_{i}{}^{\gamma \to \varepsilon }$ represents the calculated Gibbs energy change, $x_{j}$ represents the Mole friction of all the elements excluding Fe in the studied steel, and $\Omega_{\mathrm{Fej}}^{\gamma \to \varepsilon }$ is the interaction parameter of Fe and other elements.

The calculation indicated that the investigated weld metal had an increased SFE of 19.9~24.5 mJ/m^2^ at −196 °C by adding 3.9~4.2 wt. % of Ni, reducing the amount of Cr to 2.7~3.3 wt. % and Si to 0.06~0.40 wt. %. The yield strength of the weld metal at room temperature eventually reached 565 MPa, the tensile strength reached 691~882 MPa, and the cryogenic impact absorbed energy achieved 51~106 J at −196 °C. The tensile strength of the welded joints improved to 686~780 MPa, which meets the requirement of IMO mechanical properties for LNG storage tanks. It was a good example that the welded joints obtained the higher tensile strength via undermatching welding.

### 4.4. Stability of Mechanical Property by Solid Wire Welding Consumables

From Table 7 and Table 8, the mechanical properties of the weld metal are stable. One of the contributions was proposed to be the solid core of electrode and solid wire. The flux-cored welding wire may have uneven distribution of powders caused by manufacturing or transportation and bring uneven chemical composition to weld metal. Therefore, the solid core and solid wire were suggested for design of high-Mn steel welding consumables.

## 5. Conclusions

(1) For the plate with a thickness of 10 mm, the heat input of 13 ± 2 kJ/cm in the flat position for GTAW was the best welding parameter to obtain a welded joint with excellent comprehensive performance. For the plate with a thickness of 30 mm, the heat input of 15 ± 2.5 kJ/cm in the flat position for SAW was recommended.
(2) The weld metal of high-Mn austenitic steel was designed to have a similar composition to the base metal. The superior cryogenic toughness at the fusion line was obtained. The lowest cryogenic toughness value was detected at the FL + 5 mm for the plate with the thickness of 10 mm rather than at the fusion line.
(3) By design, the stacking fault energy of weld metal at a level of 19.9~24.5 mJ/m^2^ through alloying of Ni, Cr, and Si, the yield and tensile strength of the weld metal gained the requirement of ≥400 MPa and ≥660 MPa, respectively. Although the strength of the weld metal was lower than the base metal, the tensile strength of the welded joints reached 686~780 MPa.
(4) The microstructure of the weld metal and the HAZ were composed of full austenite. No phase transformation took place at the weld metal during solidification and continuous cooling and at the HAZ during continuous cooling by welding thermal cycle. Therefore, the hardness of the welded joints is less than 280 HV.
(5) The mechanical properties of high-Mn steel-welded joints had relatively stable levels. This was attributed to the solid core of electrode and solid welding wires.

## Figures and Tables

**Table 1:** Welding process approval test items and related evaluation indicators.

Test Item	Performance Requirement
Yield strength	Weld metal: ≥400 MPa
Tensile strength	Weld metal and Welded joint: ≥660 MPa
Total elongation	Weld metal: ≥22.0%
V-notch Charpy impact test	Average impact absorbed energy at −196 °C: ≥27 J
Bending test	Cracks no longer than 3 mm on the tensile surface at 180° bending angle
Flaw detection of the weld cross-section	No incomplete fusion, incomplete joint penetration, crack, porosity, and slag inclusions
Hardness	≤350 HV

**Table 2:** Chemical composition of the base material (wt. %).

C	Si	Mn	Cr	Cu	P	S
0.35~0.45	0.20~0.25	24.50~25.80	3.00~5.00	0.30~0.70	≤0.030	≤0.010

**Table 3:** Mechanical proprieties of the base material.

Yield Strength (MPa)	Tensile Strength (MPa)	Total Elongation (%)	V-Notch Charpy Impact Absorbed Energy at −196 °C (J)
402	852	56.5	161

**Table 4:** Welding parameters.

Plate No.	Welding Method and Position	Diameter of Core/Wire (mm)	Current Polarity	Current (A)	Voltage (V)	Travel Speed (cm/min)	Heat Input (kJ/cm)
SM-1	SMAW 1G	3.2	DCEP	92	22~26	10	16 ± 2
SM-3	SMAW 3G	3.2	DCEP	92	20~26	7	18 ± 2.5
SM-4	SMAW 4G	2.5	DCEP	70	21~24	6	16 ± 1
GT-1	GTAW 1G	2.5	DCEP	160	11~14	9	13 ± 2
SA-1	SAW 1G	3.2	DCEP	430	25~33	50	15 ± 2
SA-1-15	SAW 1G	3.2	DCEP	430	25~33	50	15 ± 2
SA-1-30	SAW 1G	3.2	DCEP	550	29~33	33	30 ± 2.5

**Table 5:** Chemical composition of the investigated weld metals (wt. %).

C	Si	Mn	Cr	Ni	P	S
0.36~0.40	0.06~0.40	23.2~24.5	2.7~3.3	3.9~4.2	<0.010	<0.010

**Table 6:** Macroscopic examination results of the investigated welded joints.

Sample No.	Crack	Incomplete Fusion	Incomplete Joint Penetration	Porosity	Slag Inclusions
SM-1	No	No	No	No	No
SM-3	No	No	No	No	No
SM-4	No	No	No	No	No
GT-1	No	No	No	No	No
SA-1	No	No	No	No	No
SA-1-15	No	No	No	No	No
SA-1-30	No	No	No	No	No

**Table 7:** Tensile properties of the weld metals and the welded joints at room temperature.

Plate No.	Plate Thickness (mm)	Sample	Yield Strength (MPa)	Tensile Strength (MPa)	Elongation (%)
SM-1	10	WM	447	760	43.9
		WJ	—	764 ± 9	—
SM-3	10	WM	438	739	39.3
		WJ	—	699 ± 1	—
SM-4	10	WM	440	717	30.9
		WJ	—	686	—
GT-1	10	WM	469	758	50.3
		WJ	—	772 ± 8	—
SA-1	10	WM	392	691	51.5
		WJ	—	708 ± 8	—
SA-1-15	30	WM	549	882	44.5
		WJ	—	764 ± 8	—
SA-1-30	30	WM	565	764	33.0
		WJ		754 ± 11	

**Table 8:** Cryogenic V-notch Charpy impact absorbed energy at −196 °C.

Plate No.	WM (J)			FL (J)			FL + 1 (J)			FL + 3 (J)			FL + 5 (J)		
SM-1	78	84	69	56	45	51	60	62	60	45	51	47	45	45	45
	77 ± 6 *			51 ± 4 *			61 ± 1 *			48 ± 2 *			45 ± 0 *		
SM-3	56	65	74	44	40	54	51	54	55	45	42	50	37	44	42
	65 ± 7 *			46 ± 8 *			53 ± 2 *			46 ± 3 *			41 ± 3 *		
SM-4	51			45			50			50			42		
GT-1	82	87	90	65	65	75	61	61	60	59	65	54	57	57	56
	86 ± 3 *			68 ± 5 *			61 ± 0 *			59 ± 4 *			57 ± 0 *		
SA-1	91	81	106	81	90	76	62	65	60	56	56	57	54	46	51
	93 ± 10 *			82 ± 6 *			62 ± 2 *			56 ± 0 *			50 ± 3 *		
SA-1-15	81	84	72	74	81	75	73	78	78	86	86	78	92	96	99
	79 ± 5 *			77 ± 3 *			76 ± 2 *			83 ± 4 *			96 ± 3 *		
SA-1-30	106	96	96	90	102	106	85	90	88	87	80	89	103	93	89
	99 ± 4 *			99 ± 7 *			88 ± 2 *			85 ± 4 *			95 ± 6 *		

* Average and standard deviation.

**Table 9:** Results of transverse side bend test.

Plate No.	Sample Number	Thickness (mm)	Bend Diameter (mm)	Bend Angle (°)	Result
SM-1	4	10	40	180	No opening defects after test
SM-3	4	10	40	180	No opening defects after test
SM-4	4	10	40	180	No opening defects after test
GT-1	4	10	40	180	No opening defects after test
SA-1	4	10	40	180	No opening defects after test
SA-1-15	4	10	40	180	No opening defects after test
SA-1-30	4	10	40	180	No opening defects after test

**Table 10:** Summary of the mechanical properties of the welded joint (WJ) and the weld metal (WM).

Test Item	Requirement	SM-1	SM-3	SM-4	GT-1	SA-1	SA-1-15	SA-1-30
Yield strength of WM	≥400 MPa	447 MPa	438 MPa	440 MPa	469 MPa	392 MPa	549 MPa	565 MPa
Tensile strength of WM	≥660 MPa	760 MPa	739 MPa	717 MPa	758 MPa	691 MPa	882 MPa	764 MPa
Total elongation of WM	≥22%	43.9%	39.3%	30.9%	50.3%	51.5%	44.5%	33.0%
Tensile strength of WJ	≥660 MPa	764 ± 9 MPa	699 ± 1 MPa	686 MPa	722 ± 8 MPa	708 ± 8 MPa	764 ± 8 MPa	754 ± 11 MPa
V-notch Charpy impact test	≥27 J	45–84 J	37–74 J	42–52 J	54–90 J	46–106 J	72–99 J	85–106 J
Hardness	≤350 HV	≤280 HV	≤256 HV	≤253 HV	≤264 HV	≤248 HV	≤275 HV	≤263 HV

## Data Availability

Not applicable.
